# Evaluation of Non-Celiac Gluten Sensitivity in Patients with Previous Diagnosis of Irritable Bowel Syndrome: A Randomized Double-Blind Placebo-Controlled Crossover Trial

**DOI:** 10.3390/nu12030705

**Published:** 2020-03-06

**Authors:** Michele Barone, Eugenio Gemello, Maria Teresa Viggiani, Fernanda Cristofori, Caterina Renna, Andrea Iannone, Alfredo Di Leo, Ruggiero Francavilla

**Affiliations:** 1 Section of Gastroenterology, Department of Emergency and Organ Transplantation, University of Bari “Aldo Moro”, 70100 Bari, Italy; viggiani.mt@libero.it (M.T.V.); ianan@hotmail.com (A.I.); alfredo.dileo@uniba.it (A.D.L.); 2 Center for the Study and Treatment of Eating Disorders ASL/LE, 73100 Lecce, Italy; egemello@libero.it (E.G.); caterinarenna@gmail.com (C.R.); 3 Interdisciplinary Department of Medicine, Paediatric Section, University of Bari “Aldo Moro”, 70100 Bari, Italy; fernandacristofori@gmail.com (F.C.); ruggiero.francavilla@uniba.it (R.F.)

**Keywords:** gluten-free diet, FODMAP, gluten challenge

## Abstract

Background. To date, there is no reliable marker for the diagnosis of non-celiac gluten sensitivity (NCGS), which benefits from a gluten-free diet (GFD). This condition is characterized by functional gastrointestinal symptoms similar to those occurring in the course of irritable bowel syndrome (IBS). However, IBS has a higher prevalence, and often benefits from the administration of a low fermentable oligosaccharides, disaccharides, monosaccharides and polyols (FODMAP) diet. The overlap of symptoms between these two pathologies has led to an overestimation of self-made diagnosis NCGS. Aims. To better identify NCGS in subjects with a previous diagnosis of IBS. Methods. All subjects received a low FODMAP diet that was also gluten-free (low FODMAP-GFD), and those presenting an improvement of symptoms were exposed to gluten or placebo (double-blind challenge with wash-out and crossover). The response to dietary treatments was evaluated by visual analogue scale (VAS). Results. Of 30 patients (23 women, seven men, aged 42.2 ± 12.5 years, body mass index (BMI) 24.7 ± 4.1 kg/m^2^), 26 benefited from the administration of low FODMAP-GFD and were exposed to the gluten/placebo challenge. After the challenge, using an increase of visual analogue scale VAS (Δ-VAS) ≥30%, 46.1% of the patients were NCGS+. However, this percentage became only 19.2% using a different method (mean ∆-VAS score plus two standard deviations). Conclusions. FODMAP intolerance could hide the response to a challenge test with gluten for the identification of NCGS in IBS patients. A low FODMAP-GFD followed by gluten/placebo challenge is able to identify patients with NCGS better. ClinicalTrials.gov registration number NCT04017585.

## 1. Introduction

“Non-Celiac Gluten Sensitivity” (NCGS) is a syndrome characterized by intestinal and extra-intestinal symptoms related to the ingestion of food containing gluten in subjects in whom celiac disease and wheat allergy have been excluded [1]. To date, there is no reliable marker for the diagnosis of NCGS, and the only way to make a diagnosis relies on a challenge with gluten that meets the Salerno criteria [1]. Moreover, the functional gastrointestinal symptoms that occur in the presence of NCGS are similar to those occurring in irritable bowel syndrome (IBS) [2], a condition that has a high prevalence in Western countries [3] and often benefits from the administration of a low fermentable oligosaccharides, disaccharides, monosaccharides and polyols (FODMAP) diet [4].

The overlap of symptoms between NCGS and the more common IBS, together with the difficulty to establish a correct diagnosis of both these conditions, have generated a growing trend to adopt a gluten-free diet (GFD) in subjects with functional gastrointestinal symptoms [5]. The popularity of gluten-free products has grown disproportionately, as demonstrated by the worldwide increase in their sales estimated to reach US $7.59 billion by 2020 [6].

In 2013, Biesiekierski et al. [7] demonstrated how essential is the dietary reduction of FODMAPs before the evaluation of the specific effects of gluten in patients with putative NCGS.

Based on the considerations mentioned above, for a more practical approach to the diagnosis of NCGS, the response to the gluten challenge should be made after the administration of a low FODMAP diet that is also completely gluten-free. This approach would not only be able to recognize patients with NCGS, but also IBS patients who are not responsive to the dietary treatments. Both the low FODMAP diet and GFD are the only dietary treatments effective in IBS patients since a generic approach based on reduced intake of insoluble fibers, alcohol, caffeine, spicy foods and fat has shown limited evidence for a beneficial role in these pathological conditions [8].

This study aimed to achieve a definitive diagnosis of NCGS in IBS patients. For this purpose, all subjects received a low FODMAP diet combined with a GFD (low FODMAP-GFD), and those presenting an improvement of symptoms during this dietary treatment were exposed to a double-blind challenge with gluten or placebo.

## 2. Materials and Methods

This paper reports a prospective randomized, double-blind placebo-controlled crossover trial to test the clinical response to gluten. The study was performed between April and September 2019 and was sponsored and coordinated by the Center for the Study and Treatment of Eating Disorders ASL/LE, Lecce, Italy in Lecce (Italy).

Our research was carried out in compliance with the Helsinki Declaration, all patients gave their informed consent, all procedures received the local ethics committee’s approval (protocol #31, 8 April 2019) and the trial was registered on ClinicalTrials.gov (registration # NCT04017585).

The inclusion criteria were a previous diagnosis of IBS based on Rome IV criteria, absence of alarm symptoms, and no use of medications for the treatment of bowel habit abnormalities in the previous 3 months [9].

Exclusion criteria were administration of a gluten-free diet in the previous six months, presence of celiac disease or wheat allergy, chronic intestinal inflammatory diseases, psychiatric disorders, major abdominal surgery (in particular intestinal resections), diabetes mellitus, previous anaphylactic episodes and pregnancy.

Before entering the diagnostic step, all patients underwent the following tests, as suggested Fasano et al. [10]: (a) antigliadin-antibodies-IgA (if not previously determined; ORGENTEC Diagnostika; Mainz, Deutschland); (b) hematological parameters including hemoglobin, serum iron, ferritin, aspartate aminotransferase and erythrocyte sedimentation rate; (c) human leukocyte antigen class II typing (DQ-CD Typing Plus; DiaGene, Palermo, Italy). In addition, for ethical reasons, duodenal biopsy was offered on voluntary basis only in patients with human leukocyte antigen DQ 2/8 (10).

The primary outcome of this study was to achieve a definitive diagnosis of NCGS in IBS patients; the secondary outcome was to recognize patients responsive to a low FODMAP diet and patients that do not respond either GFD or low FODMAP diet.

## 3. Study Design

**Figure 1:**
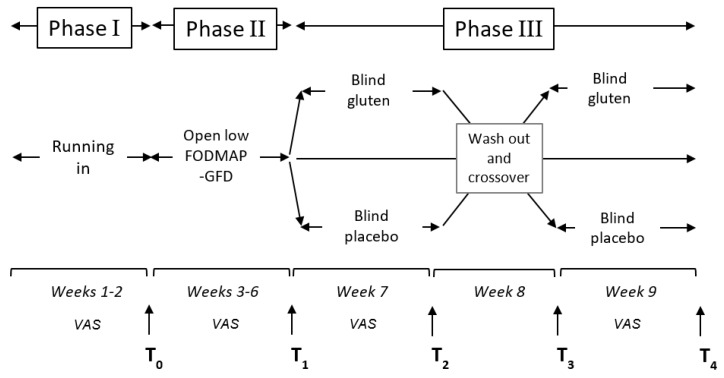
A detailed description of the three phases of the study.

The diagnostic steps included three phases (run-in, low FODMAP-gluten elimination phase, gluten challenge) (Figure 1), during which all patients were asked to complete a global daily visual analogue scale (VAS) corresponding to their perception of gastrointestinal symptoms; VAS is a 0 to 10 scale ranging from 0 (no symptom) to 10 (worst possible symptom) [1].

The run-in phase (phase 1, two weeks) aimed to assess basal symptoms while on a FODMAP-gluten-containing diet; only symptomatic patients progressed to the open diagnostic phase following a balanced normocaloric low FODMAP-GFD for a period of four weeks (phase 2: T0–T1). At the end of phase 2, the modification of symptoms was evaluated as reported by Catassi et al. [1] using a VAS score reduction of ≥30% as a significant response. In the presence of a VAS score reduction of ≥30% at the end of low FODMAP-GFD, the challenge phase (phase 3: week 7–9) was offered. Patients with a VAS score reduction <30% were considered “non-responders” and discontinued the trial.

The variation of symptoms in patients that completed phase 3 was evaluated using two different approaches. The first has been described in the Salerno criteria [1] (VAS score increase ≥30%), and the second by Di Sabatino et al. [11]. This author calculated the ∆-VAS score (VAS gluten − VAS placebo) for each patient and the mean ∆-VAS score and its standard deviation for the population under analysis and finally, a VAS score variation greater than the mean ∆-VAS score + 2SD was considered significant.

## 4. Gluten Challenge Phase

The low FODMAP-GFD responders were randomized based on a computer-generated randomization list to take gluten or placebo for 7 days (phase 3, T1–T2) (Figure 1); then, the patients from both groups started the wash-out period of one week (T2–T3) and subsequently started the final week (T3–T4) on placebo or gluten sachets.

Two sachets a day of gluten or placebo were administered. The gluten used contained 80% protein; the non-protein part was mainly made of starch (14%), fibers (2%), fat (1.5%) and ash (0.75%). Each sachet contained either 9 g of gluten or placebo (indistinguishable in appearance and texture). The amount of gluten was calculated based on the average consumption of food containing gluten (90 g of pasta/day, which corresponds to about 10 g of gluten) [12]. Each sachet contained ~0.4 g of ATIs and no more than 0.04 g of FODMAP, which is irrelevant as compared to the average daily ingestion. All patients were instructed to disperse the content in any food (avoiding water, fruit juice and milk) administered during the day. Sachets containing gluten had the same shape, dimension, indication and appearance as those containing placebo and were marked with a serial number. The blinding was validated in 10 healthy adult volunteers in a triangle test performed before the trial. The manufacturer that provided this material was independent of the study (Consorzio di Ricerca “Gian Pietro Ballatore” of Palermo, Italy, a research center focused on the study of cereals), and to ensure blindness of the investigators and patients, only the manufacturers were in a position to associate each sachet number with its content. Rice starch was chosen as the placebo to avoid Fermentable Oligosaccharides Disaccharides Monosaccharides and Polyols (FODMAP)-containing substrate. To assess compliance, medical personnel interviewed patients on a regular basis. Compliance was calculated as the percentage of returned sachets and ingested study product: a rate higher than 80% was set as the minimum for both.

## 5. Nutritional Evaluations and Adherence to the Diet

To provide a low FODMAP-GFD, an expert dietitian calculated food energy requirements based on anthropometric parameters (weight and height), age and sex, using the Harris-Benedict formula integrated by the energy index, as previously described [13]. Each subject received a balanced normocaloric diet (50% of the calories deriving from carbohydrates, 30% from lipids and 20% from proteins) of Mediterranean type, with multiple food choice [14]. The use of nutritional supplements, probiotics and prebiotics were excluded. An expert dietitian also evaluated food preferences, eating patterns and cooking methods. To evaluate adherence, participants were asked to complete a food diary at the entrance and after each phase of the study. Compliance was calculated as the percentage of returned sachets and ingested study product: a rate greater than 80% was set as the minimum for both. The dietician instructed all patients on how to fill out a detailed daily report on the food and beverages consumed.

## 6. Preparation of Low FODMAP-GFD

The gluten-free diet was obtained by permitting the consumption of only cereals or pseudo-cereals that are gluten-free, such as rice, buckwheat, corn, millet and quinoa (all naturally gluten-free foods with low FODMAP content) [15,16]. Moreover, we allowed only the consumption of vegetables and fruit poor in FODMAP, following the nutritional program that received validation to treat irritable bowel syndrome (IBS) [17]. The vegetables allowed were green beans, fennel, carrots, zucchini, pumpkin, cucumbers, celery, tomato, lettuce, fennel, endive, olive, cucumber, pumpkin, pepper and radishes, whereas fruits permitted were banana, blueberry, strawberry, raspberry, melon, white melon, pineapple, citrus fruits and kiwi [16,18].

## 7. Statistical Analysis

Normally, distributed grouped data were expressed as mean ± standard deviations (SD) and compared by *t*-test. Non-parametric grouped data were expressed as means (95% CI) and compared using the Mann–Whitney rank sum test (paired) or Wilcoxon’s signed-rank test (unpaired). Categorical variables were expressed as percentages and compared using the chi-square or Fisher’s exact test. The calculation of the risk difference estimated the response to the gluten challenge (primary outcome). The statistical significance was set at *p* < 0.05. Assuming a 30% positivity to the challenge, we estimated that 30 patients would be required for the study to have 80% power and a two-sided 5% significance level. The analyses were performed using SPSS software, version 23.0 (SPSS Inc., Chicago, IL, USA).

## 8. Results

**Figure 2:**
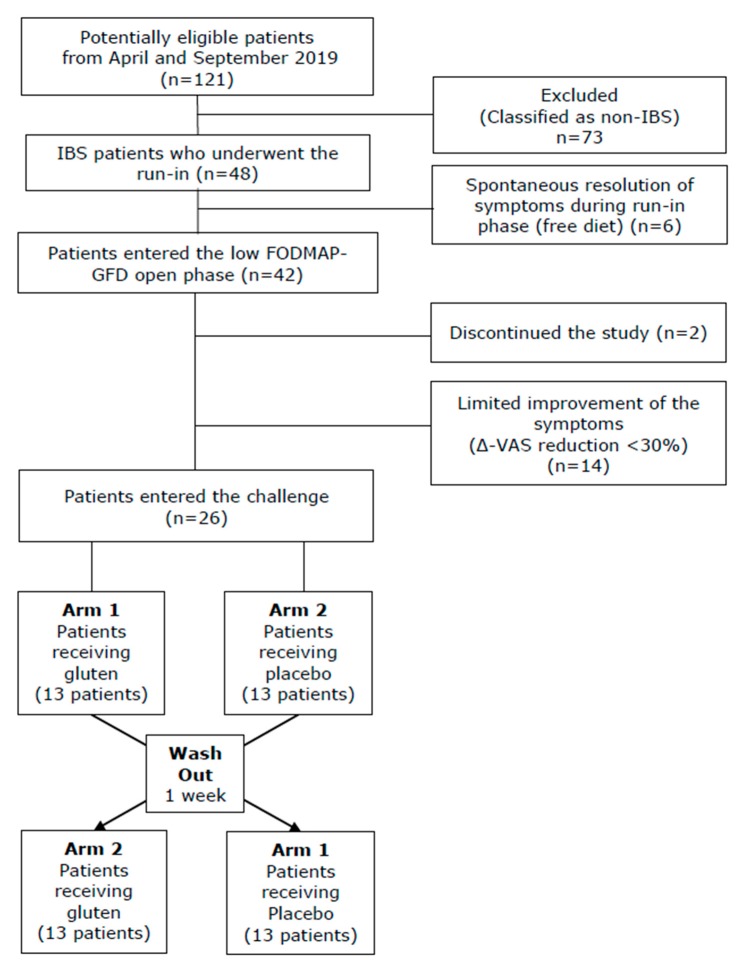
Flow diagram describing the process from assessment for eligibility through the completion of the study. IBS, irritable bowel syndrome; FODMAP, fermentable oligosaccharides, disaccharides, monosaccharides and polyols; VAS, visual analogue scale.

The flow of patients involved in the trial from assessment for eligibility through follow-up is shown in Figure 2. Overall, out of a total of 121 patients referred to our gastroenterology out-patients service for evaluation of gastrointestinal symptoms, 48 patients (39.5%) received a diagnosis of IBS, and therefore, were considered eligible. Six patients (6.5%) were excluded as they experienced a spontaneous resolution of the symptoms during the run-in phase. The remaining 42 patients proceeded to the low FODMAP-GFD open phase, with the invitation to refrain from modification to the dietetic habits. During this phase, two patients interrupted the follow-up and quit the study. Therefore, only 40 individuals completed this phase. The demographic, clinical and biochemical parameters of these patients are reported in Table 1. All patients complained abdominal pain (100%), while other symptoms were less frequent: abdominal distention (50%), alternate phases of constipation and diarrhea (38%) and chronic fatigue (29%). No differences were found among the patients who exited or continued the diagnostic trial (data not shown). Among enrolled patients, 15 carried the human leucocyte antigen predisposition.

**Figure 3:**
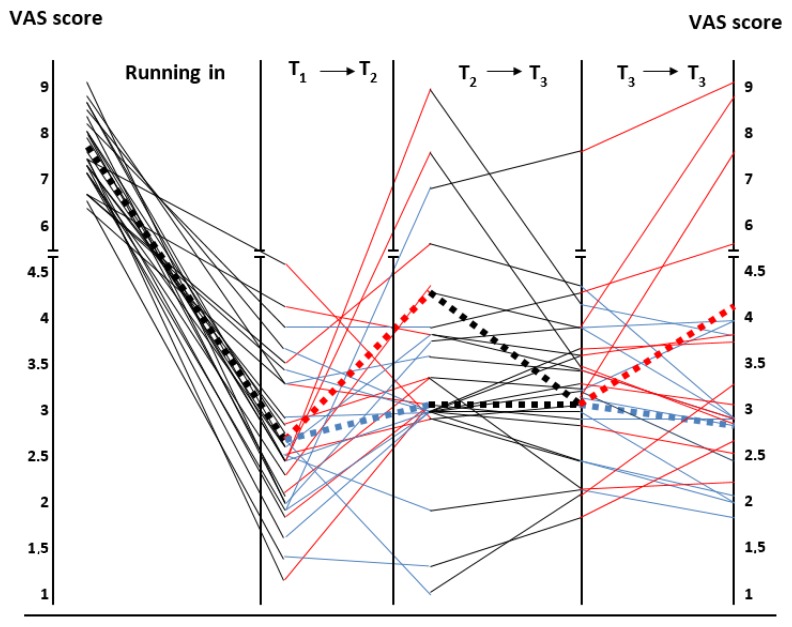
Graphical representation of the VAS score changes from the beginning to the end of the study. Black continuous lines refer to the phases with low FODMAP-GFD and red and blue continuous lines correspond to the gluten and rice challenge, respectively. The ends of each dotted line represent the mean of the VAS values obtained at the beginning and the end of each phase of the study.

Twenty-six patients (65%; 21 females) reported a significant improvement in their symptoms with this nutritional regimen as demonstrated by the assessment of VAS score before and after administration of the open low FODMAP-GFD (7.9 ± 0.8 vs. 2.7 ± 1.0, respectively; *p* = 0.001). Figure 3 describes in detail the variations of the VAS score in the relationship with the different phases of the study. The 14 patients (35%) that did not respond to the low FODMAP-GFD showed no difference compared to the responders when the parameters reported in Table 1 were considered (data not shown).

**Figure 4:**
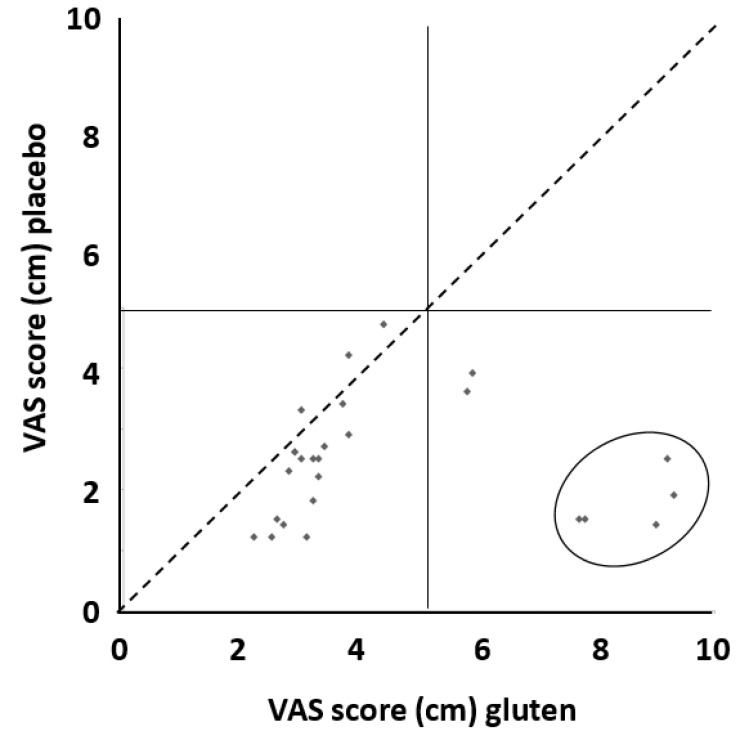
Distribution of patients according to their VAS score while on gluten and placebo. Patients closer to the diagonal dashed line had a similar response to gluten and placebo. The two patients located in the lower right square but not included in the ellipse experienced a mild degree of the overall response. Patients included in the ellipse had a significant increase in VAS score ≥5.0 cm after gluten challenge (for the calculation of this value refer to the “Materials and Methods” section). The risk of symptom worsening after gluten assumption was increased by five times (95% CI 0.63 to 39.91) compared to placebo, although such a difference did not reach statistical significance. The absolute risk difference was 15% (95% CI − 1% to 32%).

All 26 FODMAP-GFD responsive patients underwent the gluten or placebo challenge as described in the material and methods section. According to the Salerno criteria, after the administration of gluten, 12 (46.1%) patients reported a worsening of their symptoms since they had an increase of VAS score ≥30% as compared to placebo, and therefore, could be considered affected by NCGS. However, when we considered the criteria of Di Sabatino et al. for the diagnosis of NCGS, only five patients (19.2%) were found to suffer from NCGS (Figure 4). Interestingly, one patient (3.8%) increased the VAS score ≥30% when receiving the placebo after the low FODMAP-GFD open phase. However, the VAS score of this patient did not increase significantly after the switch to the gluten group. 

## 9. Discussion

NCGS is a pathological condition characterized by intestinal and/or extra-intestinal symptoms that are related to the ingestion of gluten in subjects, prevalently women [19], that are not affected by either CD or wheat allergy (WA). This condition is drawing more and more interest considering the apparent alarming increase of its incidence in the general population that is mostly due to self-diagnosed gluten intolerance leading to the adoption of a GFD without any medical prescription [20]. The prevalence of this condition is unknown; the most extensive series of self-reported gluten avoidance studies ranges from 0.5 to 13% of the general population [21,22] and according to a recent population-based study, between 2009 and 2014, there was a significant increase in the prevalence of people who avoid gluten, ranging from 0.5% to 1.7% with a parallel growth in the overall sales of the gluten-free industry [23].

Whether NCGS represents a distinct entity is still controversial since there are no diagnostic biomarkers or a classic histologic lesion. Moreover, scientific evidence suggests that gluten-related adverse reactions may be due to wheat components other than gluten or to dietary components (FODMAP), which are nondigestible carbohydrates also present in high concentrations in wheat flour. Finally, gluten does not induce gastrointestinal symptoms in healthy volunteers [24].

In a double blind placebo controlled study, Biesiekierski et al. (7) reported that patients with NCGS do not exhibit statistically significant effects after gluten has been added to the diet by reducing foods high in FODMAP at the same time, indicating that symptoms in patients with NCGS may be due to FODMAP rather than gluten. Skodje et al. [25] conducted a study involving 59 GFD patients divided into three groups receiving: gluten-containing diet (5.7 g), fructans (2.1 g) and placebo, respectively. After 7 days, patients underwent a washout period and were crossed over into a different group, until they completed the three challenges. These investigators found that the average symptom levels in the FODMAP community were significantly higher in fructans than in the gluten and placebo categories. In comparison, they showed that a diet rich in FODMAPs induced greater fatigue relative to the placebo and gluten classes.

To show that gluten is a causal factor in NCGS patients, Rosinach et al. [26] conducted a study in which 18 HLA-DQ2/8 + patients with GI symptoms, negative coeliac serology and gluten-dependent lymphocytic enteritis were assigned to gluten (20 gr) or placebo groups; no FODMAP content was present in both types of sachets. Ninety-one percent of patients experienced clinical relapse during gluten challenge versus 28.5% after placebo, indicating that gluten is the trigger of symptoms in a subgroup of patients meeting the NCGS diagnostic criteria.

Therefore, it is still unclear which component of the diet is the causal agent for GI symptoms. Indeed, Carroccio et al. [27] gathered and analyzed evidence from 200 patients and found that nearly 90% of wheat-free diet (WFD) patients were distinguished by a significant improvement in IBS symptoms. The authors concluded that wheat rather than gluten is the causal agent of this chronic disorder, and therefore, patients should be correctly identified and treated with a wheat-free diet.

Whatever the proposed elimination diet is, all have their drawbacks. GFD is more expensive than a gluten-containing diet and can also induce some food deficiencies due to a lower consumption of fibers, a reduced vitamin intake (Vitamin D, Vitamin B12 and folate), some mineral deficiencies (iron, zinc, magnesium and calcium) and increased lipid intake [28,29]. A low FODMAP diet may cause some nutrient deficiencies, and therefore, it should be followed for a relatively short period under the supervision of an experienced dietitian [2]. Finally, both diets are responsible for unfavorable alterations of the microbiota and metabolome, such as a reduction of Bifidobacteria or a substantially lower total bacterial load [30].

All the above-mentioned considerations lead to the conclusion that a correct dietary treatment in IBS patients requires a definitive diagnosis of putative food intolerance before any dietary manipulation. In the present study, we used a combination of a low FODMAP diet along with a GFD as a practical approach to recognizing three categories of IBS patients. The first consists of patients who are not responsive to both dietary treatments, the second one consists of patients responsive to a low FODMAP diet (patients that improved during the low FODMAP-GFD open phase not susceptible to the gluten challenge) and, finally, the last category consists of patients with NCGS.

In our research, the first group of IBS patients included four (15.4%) subjects not responsive to diet modification who could benefit from medical treatment targeting altered GI motility, visceral hyperalgesia, increased intestinal permeability, immune activation and disturbances in brain-gut function [9]. As regards the second and third group of IBS patients suffering from either FODMAP intolerance or NCGS, our results changed according to the criterion adopted for the diagnosis. Adopting the Salerno criteria, 12 (46.1%) patients suffered NCGS; while using the Di Sabatino criteria, NCGS was diagnosed in five (19.2%) subjects. The remaining 21 patients were recognized as FODMAP intolerant.

Which criteria are the best to use for the diagnosis of NCGS after a gluten challenge is still controversial. However, the adoption of the Di Sabatino criteria allowed us to replicate our experience in the pediatric population, demonstrating a significant difference in the number of NCGS diagnoses according to the two criteria [31]. Unfortunately, this diagnostic approach is not routinely applicable; therefore, we also evaluated our results applying the criteria used in a previous large multicenter Italian study by Elli et al. [32], who found a prevalence of NCGS in IBS patients of about 14% using a Δ-VAS cut-off score of 3 cm. When we used Elli’s approach, we found that four patients, corresponding to 15.4% of our cohort, were affected by NCGS, which is a result similar to that reported by Elli. Moreover, our results are further supported by the data summarized in a comprehensive review of randomized controlled trials showing a clinical response up to 80% in IBS patients receiving a low FODMAP diet [33].

One of the main questions is how to determine whether a re-challenge should be considered positive. We found a vast difference in positivity when applying the Salerno Criteria or the approach suggested by Di Sabatino et al. The two results may provide upper and lower values for the prevalence of this condition since all patients identified by the Di Sabatino approach were included in those identified using the Salerno criteria. The Salerno criteria have the clear advantage of being applicable in daily clinical practice, since they are not based on calculations that require a reference population as for the Di Sabatino approach. Although methodological flaws related to the heterogeneity of the population under investigation, outcome measures and challenge design (the type of protein, the dose of gluten, delivery mode and duration of wash-out period) were present, all trials demonstrated that the overwhelming majority of patients who present gluten-related symptoms are not affected by NCGS.

The strength of our study is based on two main aspects: (a) combining a low FODMAP and a gluten-free diet, it was easy to recognize non-responders to dietary treatments that would benefit of a non-dietetic approach and (b) the adoption of a low FODMAP-GFD diet reduced the “background noise” due to a possible coexistence of both types of food intolerance. The short duration (one week) of the wash-out and gluten challenge allowed us to balance compliance and efficacy, since symptoms related to gluten usually arise soon after gluten intake [20] and the shortening of challenge reduces the risk of drop-outs.

Our study has some limitations consisting in the number of patients (mainly due to the short duration of the enrolment period) and the impossibility to exclude cases of seronegative celiac disease among our NCGS patients, an event, however, that is hardly possible given the rarity of the condition [34]. Lastly, the overwhelming effect of FODMAP reduction in our patients would have needed an evaluation of the re-introduction of FODMAP containing food (“fructan arm”) that we believe is a necessary step in the clinical management of these patients. Finally, the adoption of the Di Sabatino criteria is not routinely applicable; however, our study not only confirms the reliability of this approach, but supports the method used by Elly et al., which represents a practical and easy method for the diagnosis of NCGS.

In conclusion, FODMAP intolerance could hide the response to a challenge test with gluten for the identification of NCGS in IBS patients. A low FODMAP-GFD followed by gluten/placebo challenge would better identify patients with NCGS. We believe that our study might be useful to clinicians treating patients with IBS in guiding the type of approach in case of suspected NCGS and reducing the first-line use of various drugs and supplements, with clear direct and indirect economic advantages.

## Figures and Tables

**Table 1:** Demographic, clinical and biochemical characteristics of patients who completed phase 1 (T_0_–T_1_) of the study.

Variables	Values at T_0_
N. of subjects	40 (100%)
male/female°	10/30
age (years)	40.5 ± 12.5
BMI	24.7 ± 4.1
Abdominal pain (%)	100
Abdominal distention (%)	50
Constipation/diarrhea (%)	38
Fatigue (%)	29
Hemoglobin (g/dL)	14.5 ± 0.8
Ferritin (ng/mL)	183.1 ± 43.6
AST (U/mL)	23.6 ± 3.9

Age, BMI, and biochemical values were expressed as mean ± SD. Hemoglobin, ferritin and AST normal range values are 13 g/dL, 50–250 ng/mL and 10–30 U/mL, respectively.

## Abbreviations

Celiac Disease (CD), Double-Blind Placebo-Controlled (DBPC), Fermentable Oligosaccharides Disaccharides Monosaccharides And Polyols (FODMAP), Gluten-Free Diet (GFD), Irritable Bowel Syndrome (IBS), Non-Celiac Gluten Sensitivity (NCGS), Visual Analogue Scale (VAS), Wheat Allergy (WA).

## References

[1] Catassi C., Elli L., Bonaz B., Bouma G., Carroccio A., Castillejo G. (2015). Diagnosis of Non Celiac Gluten Sensitivity (NCGS): The Salerno Experts Criteria. Nutrients.

[2] Catassi C., Alaedini A., Bojarski C., Bonaz B., Bouma G., Carroccio A., Castillejo G., De Magistris L., Dieterich W., Di Liberto D. (2017). The Overlapping Area of Non-Celiac Gluten Sensitivity (NCGS) and Wheat-Sensitive Irritable Bowel Syndrome (IBS): An Update. Nutrients.

[3] Van den Houte K., Carbone F., Pannemans J., Corsetti M., Fischler B., Piessevaux H., Tack J. (2019). Prevalence and impact of self-reported irritable bowel symptoms in the general population. United Eur. Gastroenterol. J..

[4] Whelan K., Martin L.D., Staudacher H.M., Lomer M.C.E. (2018). The low FODMAP diet in the management of irritable bowel syndrome an evidence-based review of FODMAP restriction, reintroduction and personalisation in clinical practice. J. Hum. Nutr. Diet.

[5] Jones A.L. (2017). The Gluten-Free Diet: Fad or Necessity?. Diabetes Spectr..

[6] The Lancet Gastroenterology Hepatology (2016). Gluten: Going against the grain?. Lancet Gastroenterol. Hepatol..

[7] Biesiekierski J.R., Peters S.L., Newnham E.D., Rosella O., Muir J.G., Gibson P.R. (2013). No effects of gluten in patients with self-reported non-celiac gluten sensitivity after dietary reduction of fermentable, poorly absorbed, short-chain carbohydrates. Gastroenterology.

[8] Cozma-Petruţ A., Loghin F., Miere D., Dumitraşcu D.L. (2017). Diet in irritable bowel syndrome: What to recommend, not what to forbid to patients!. World J. Gastroenterol..

[9] Lacy B.E., Mearin F., Chang L., Chey W.D., Lembo A.J., Simren M., Spiller R. (2016). Bowel Disorders. Gastroenterology.

[10] Fasano A., Catassi C. (2012). Clinical practice. Celiac disease. N. Engl. J. Med..

[11] Di Sabatino A., Volta U., Salvatore C., Biancheri P., Caio G., De Giorgio R., Di Stefano M., Corazza G.R. (2015). Small Amounts of Gluten in Subjects with Suspected Nonceliac Gluten Sensitivity: A Randomized, Double-Blind, Placebo-Controlled, Cross-Over Trial. Clin. Gastroenterol. Hepatol..

[12] Fois S., Campus M., Piu P.P., Siliani S., Sanna M., Roggio T., Catzeddu P. (2019). Fresh Pasta Manufactured with Fermented Whole Wheat Semolina: Physicochemical, Sensorial, and Nutritional Properties. Foods.

[13] Barone M., Viggiani M.T., Anelli M.G., Fanizzi R., Lorusso O., Lopalco G., Cantarini L., Di Leo A., Lapadula G., Iannone F. (2018). Sarcopenia in Patients with Rheumatic Diseases: Prevalence and Associated Risk Factors. J. Clin. Med..

[14] Silva D., Moreira R., Beltrão M., Sokhatska O., Montanha T., Pizarro A., Garcia-Larsen V., Villegas R., Delgado L., Moreira P. (2019). What Is the Effect of a Mediterranean Compared with a Fast Food Meal on the Exercise Induced Adipokine Changes? A Randomized Cross-Over Clinical Trial. PLoS ONE.

[15] Rai S., Kaur A., Chopra C.S. (2018). Gluten-Free Products for Celiac Susceptible People. Front. Nutr..

[16] Roncoroni L., Elli L., Doneda L., Bascuñán K.A., Vecchi M., Morreale F., Scricciolo A., Lombardo V., Pellegrini N. (2018). A Retrospective Study on Dietary FODMAP Intake in Celiac Patients Following a Gluten-Free Diet. Nutrients.

[17] Halmos E.P., Power V.A., Shepherd S.J., Gibson P.R., Muir J.G. (2014). A diet low in FODMAPs reduces symptoms of irritable bowel syndrome. Gastroenterology.

[18] Gibson P.R., Shepherd S.J. (2010). Evidence-based dietary management of functional gastrointestinal symptoms: The FODMAP approach. J. Gastroenterol. Hepatol..

[19] Camilleri M. (2020). Sex as a Biological Variable in Irritable Bowel Syndrome. Neurogastroenterol. Motil..

[20] Losurdo G., Principi M., Iannone A., Amoruso A., Ierardi E., Di Leo A., Barone M. (2018). Extra-intestinal manifestations of non-celiac gluten sensitivity: An expanding paradigm. World J. Gastroenterol..

[21] Molina-Infante J., Santolaria S., Sanders D., Fernández-Bañares F. (2015). Systematic review: Noncoeliac gluten sensitivity. Aliment. Pharmacol. Ther..

[22] Cabrera-Chávez F., Granda-Restrepo D.M., Arámburo-Gálvez J.G., Franco-Aguilar A., Magaña-Ordorica D., de Jesús Vergara-Jiménez M., Ontiveros N. (2016). Self-Reported Prevalence of Gluten-Related Disorders and Adherence to Gluten-Free Diet in Colombian Adult Population. Gastroenterol. Res. Pract..

[23] U.S Gluten-Free Foods Market—Statistics & Facts. https://www.statista.com/topics/2067/gluten-freefoodsmarket/.

[24] Croall I.D., Aziz I., Trott N., Tosi P., Hoggard N., Sanders D.S. (2019). Gluten Does Not Induce Gastrointestinal Symptoms in Healthy Volunteers: A Double-Blind Randomized Placebo Trial. Gastroenterology.

[25] Skodje G.I., Sarna V.K., Minelle I.H., Rolfsen K.L., Muir J.G., Gibson P.R., Veierød M.B., Henriksen C., Lundin K.E.A. (2018). Fructan, Rather Than Gluten, Induces Symptoms in Patients with Self-Reported Non-Celiac Gluten Sensitivity. Gastroenterology.

[26] Rosinach M., Fernández-Bañares F., Carrasco A., Ibarra M., Temiño R., Salas A., Esteve M. (2016). Double-Blind Randomized Clinical Trial: Gluten versus Placebo Rechallenge in Patients with Lymphocytic Enteritis and Suspected Celiac Disease. PLoS ONE.

[27] Carroccio A., D’Alcamo A., Iacono G., Soresi M., Iacobucci R., Arini A., Geraci G., Fayer F., Cavataio F., La Blasca F. (2017). Persistence of Nonceliac Wheat Sensitivity, Based on Long-term Follow-up. Gastroenterology.

[28] Barone M., Della Valle N., Rosania R., Facciorusso A., Trotta A., Cantatore F.P., Falco S., Pignatiello S., Viggiani M.T., Amoruso A. (2016). A comparison of the nutritional status between adult celiac patients on a long-term, strictly gluten-free diet and healthy subjects. Eur. J. Clin. Nutr..

[29] Vici G., Belli L., Biondi M., Polzonetti V. (2016). Gluten free diet and nutrient deficiencies: A review. Clin. Nutr..

[30] Olivares M., Castillejo G., Varea V., Sanz Y. (2014). Double-blind, randomised, placebo-controlled intervention trial to evaluate the effects of Bifidobacterium longum CECT 7347 in children with newly diagnosed coeliac disease. Br. J. Nutr..

[31] Francavilla R., Cristofori F., Verzillo L., Gentile A., Castellaneta S., Polloni C., Giorgio V., Verduci E., D’Angelo E., Dellatte S. (2018). Randomized Double-Blind Placebo-Controlled Crossover Trial for the Diagnosis of Non-Celiac Gluten Sensitivity in Children. Am. J. Gastroenterol..

[32] Elli L., Tomba C., Branchi F., Roncoroni L., Lombardo V., Bardella M.T., Ferretti F., Conte D., Valiante F., Fini L. (2016). Evidence for the Presence of Non-Celiac Gluten Sensitivity in Patients with Functional Gastrointestinal Symptoms: Results from a Multicenter Randomized Double-Blind Placebo-Controlled Gluten Challenge. Nutrients.

[33] Staudacher H.M., Whelan K. (2017). The Low FODMAP Diet: Recent Advances in Understanding Its Mechanisms and Efficacy in IBS. Gut.

[34] Volta U., Caio G., Boschetti E., Giancola F., Rhoden K.J., Ruggeri E., Paterini P., De Giorgio R. (2016). Seronegative celiac disease: Shedding light on an obscure clinical entity. Dig. Liver Dis..

